# Syntheses, crystal structures, Hirshfeld surface analyses and crystal voids of 1-(4-bromo­phen­yl)-2,2-di­chloro­ethan-1-one and 2,2-di­bromo-1-(*p*-tol­yl)ethan-1-one

**DOI:** 10.1107/S205698902500012X

**Published:** 2025-01-14

**Authors:** Atash V. Gurbanov, Firudin I. Guseinov, Aida I. Samigullina, Tuncer Hökelek, Khudayar I. Hasanov, Tahir A. Javadzade, Alebel N. Belay

**Affiliations:** a Excellence Center, Baku State University, Z. Xalilov Str. 23, AZ 1148 Baku, Azerbaijan; b Centro de Quimica Estrutural, Instituto Superior Tecnico, Universidade de Lisboa, Av. Rovisco Pais, 1049-001 Lisbon, Portugal; c Kosygin State University of Russia, 117997 Moscow, Russian Federation; d N. D. Zelinsky Institute of Organic Chemistry, Russian Academy of Sciences, 119991 Moscow, Russian Federation; e Hacettepe University, Department of Physics, 06800 Beytepe-Ankara, Türkiye; f Azerbaijan Medical University, Scientific Research Centre (SRC), A. Kasumzade Str. 14, AZ 1022 Baku, Azerbaijan; g Department of Chemistry and Chemical Engineering, Khazar University, Mahzati Str. 41, AZ 1096 Baku, Azerbaijan; h Department of Chemistry, Bahir Dar University, PO Box 79, Bahir Dar, Ethiopia; Institute of Chemistry, Chinese Academy of Sciences

**Keywords:** crystal structure, non-covalent inter­actions, hydrogen bond

## Abstract

The asymmetric units of the compounds, C_8_H_5_BrCl_2_O (I), and C_9_H_8_Br_2_O (II), contain two and one crystallographically independent mol­ecules, respectively. In compound (I), the planar rings are oriented at a dihedral angle of 13.23 (8)°. In crystals of both compounds, inter­molecular C—H⋯O hydrogen bonds link the mol­ecules into infinite chains along the *b*-axis direction. In crystal of (I), there are π–π inter­actions between the centroids of the parallel rings with centroid-to-centroid distances of 3.5974 (14), 3.6178 (16) and 3.9387 (16) Å while neither π–π nor C—H⋯ π(ring) inter­actions are present in (II). The Hirshfeld surface analyses of the crystal structures indicate that the most important contributions for the crystal packings are from H⋯Cl/Cl⋯H (27.5%), H⋯O/O⋯H (15.0%), H⋯Br/Br⋯H (10.2%) and H⋯H (9.0%) for (I) and H⋯Br/Br⋯H (36.1%), H⋯H (22.2%), H⋯O/O⋯H (14.1%) and H⋯C/C⋯H (13.9%) for (II). Hydrogen bonding and van der Waals inter­actions are the dominant inter­actions in the crystal packings. The volumes of the crystal voids and the percentages of free spaces in the unit cells were calculated to 111.55 Å^3^ and 12.27% for (I) and 63.37 Å and 6.69% for (II), showing that no large cavities are present in either structure.

## Abstract 2

The asymmetric units of the compounds, (I) and (II), contain two and one crystallographically independent mol­ecules, respectively. In crystals of (I) and (II), inter­molecular C—H⋯O hydrogen bonds link the mol­ecules into infinite chains along the *b*-axis direction. In crystal of (I), there are π–π inter­actions between the centroids of the parallel rings while neither π–π nor C—H⋯ π(ring) inter­actions are present in (II).

## 1. Chemical context

α-Haloketones are useful synthetic building blocks for the syntheses of pharmacologicals as well as complex organic mol­ecules (Erian *et al.*, 2003 ▸). In fact, the existence of two adjacent electrophilic centres, namely the α-halocarbon and carbonyl group, transforms these reactive carbonyl compounds into highly valuable building blocks for the construction of more complex structures (Guseinov *et al.*, 2006 ▸, 2017 ▸, 2020 ▸; Khalilov *et al.*, 2024 ▸). Over the past few decades, substantial advances have been made in the syntheses of these industrially relevant building blocks and synthetic precursors (Ma *et al.*, 2021 ▸; Mahmoudi *et al.*, 2017 ▸; Mizar *et al.*, 2012 ▸). Efforts have focused on rendering the synthetic protocols greener, more effective and versatile. Not only electron-withdrawing properties, but the halogen-bond-donor ability of the halogen atom(s) of α-haloketones can dictate their reactivity and other functional properties (Gurbanov *et al.*, 2022 ▸). For instance, recently we showed that the reaction of α,α-dihalo-β-oxo­aldehydes with di­amino­furazan at room temperature in an aceto­nitrile solution yields 20-membered macrocycles and *N*-(4-amino-1,2,5- oxo­diazol-3-yl)formamide (Guseinov *et al.*, 2024 ▸). Herein, we found that when this reaction is carried out in a chloro­form solution and at 353 K, both α-haloketones namely 1-(4-bromo­phen­yl)-2,2-di­chloro­ethan-1-one (I) and 2,2-di­bromo-1-(*p*-tol­yl)ethan-1-one (II) and *N*-(4-amino-1,2,5-oxa­diazol-3-yl) formamide are formed. Herein, we have report on the syntheses and mol­ecular and crystal structures of compounds (I) and (II) together with analyses of the Hirshfeld surfaces and crystal voids.
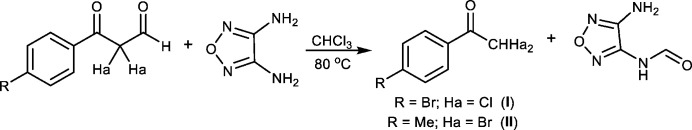


## 2. Structural commentary

**Figure 1:**
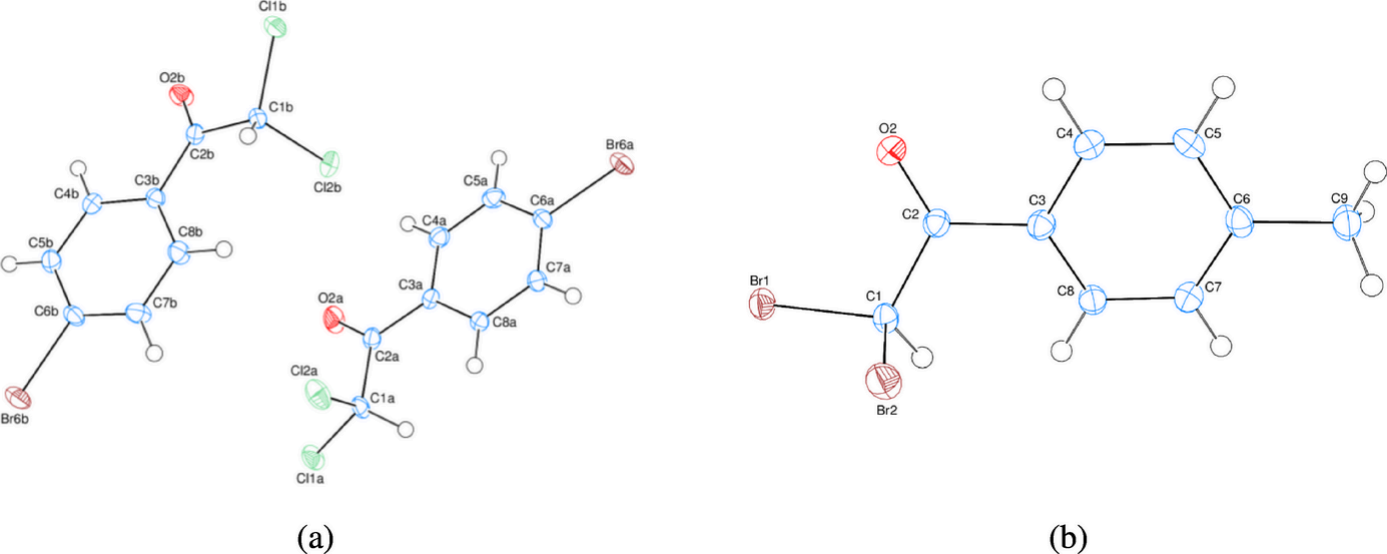
The asymmetric units of compounds (*a*) (I) and (*b*) (II) with atom-numbering schemes and 50% probability ellipsoids.

The asymmetric units of compounds (I) and (II) contains two and one crystallographically independent mol­ecules, respectively (Fig. 1 ▸). In compound (I), the planar, *A* (C3*A*–C8*A*) and *B* (C3*B*–C8*B*) rings are oriented at a dihedral angle of 13.23 (8)°. Atoms Br6*A*, C2*A*, C1*A*, O2*A* and Br6*B* and C2*B* are 0.0116 (3), 0.023 (3), −0.004 (3), 0.045 (2) Å and −0.0083 (3), −0.032 (3) Å, respectively, away from the best least-squares planes of the *A* and *B* rings. In compound (II), atoms Br1, C2 and C9 are 0.0426 (3), 0.058 (3) and 0.041 (3) Å, respectively, away from the best least-squares plane of ring *A* (C3–C8). All bond lengths and angles are normal in both compounds.

## 3. Supra­molecular features

**Figure 2:**
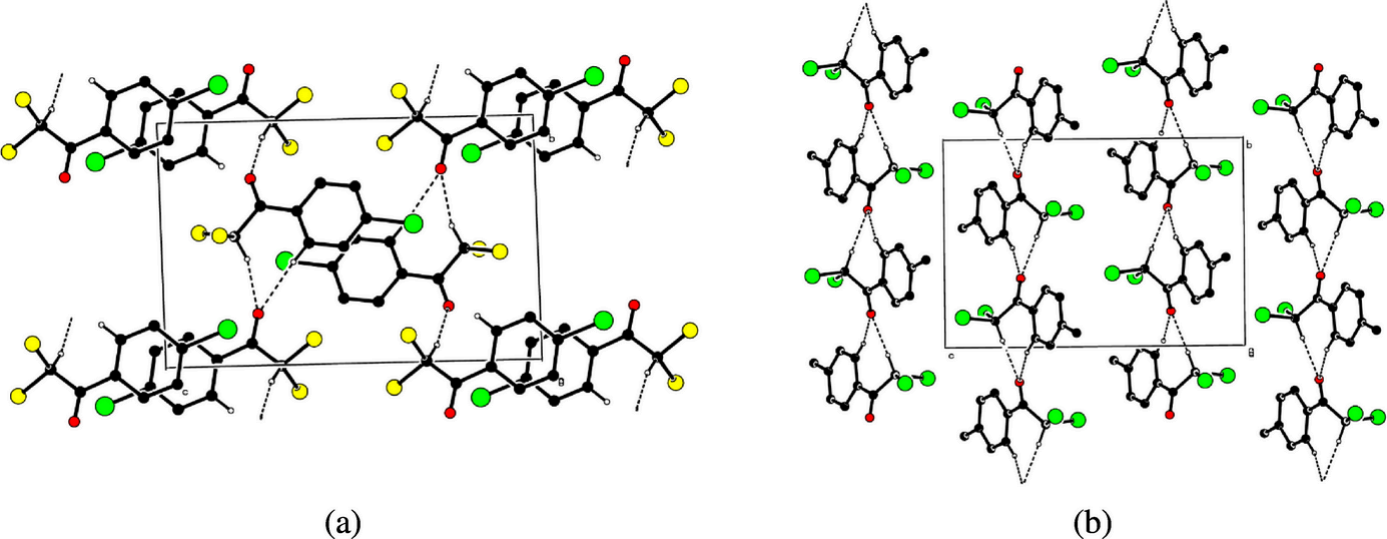
Partial packing diagrams for compounds (*a*) (I) and (*b*) (II). Inter­molecular C—H⋯O hydrogen bonds are shown as dashed lines. H atoms not involved in these inter­actions have been omitted for clarity.

In the crystals of both compounds, inter­molecular C—H⋯O hydrogen bonds (Tables 1 ▸ and 2 ▸) link the mol­ecules into infinite chains along the *b*-axis direction (Fig. 2 ▸). In crystal of (I), there are π–π inter­actions between the centroids of parallel *A* (C3*A*–C8*A*) rings and parallel *B* (C3*B*–C8*B*) rings with centroid-to-centroid distances of 3.5974 (14) Å for the *A* rings and 3.6178 (16) and 3.9387 (16) Å for the *B* rings. No such inter­actions occur in (II).

## 4. Hirshfeld surface analysis

**Figure 3:**
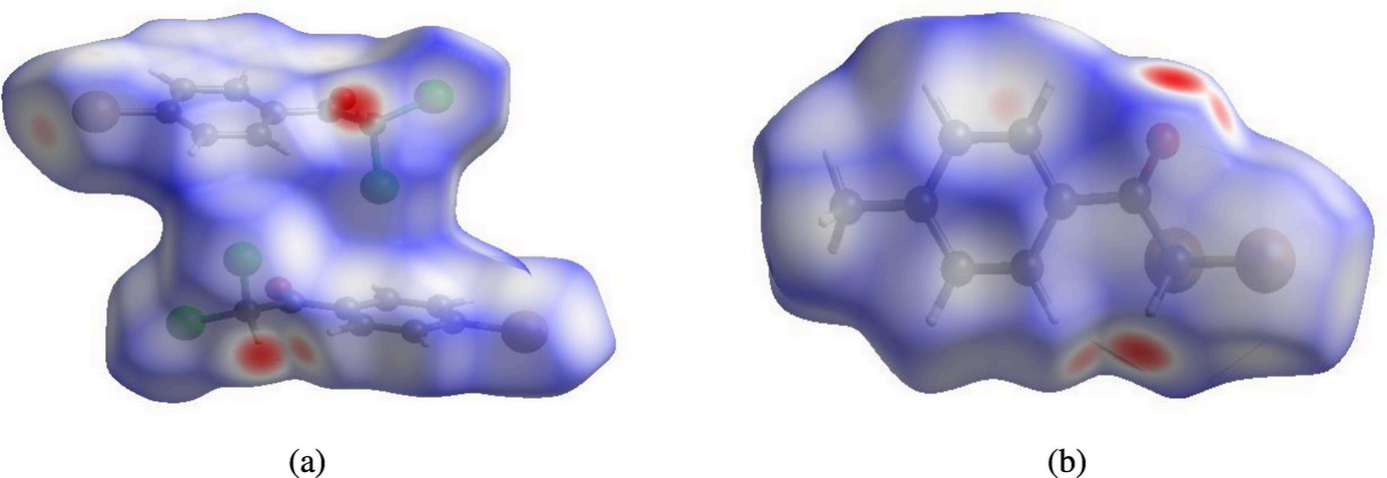
Views of the three-dimensional Hirshfeld surfaces of compounds (*a*) (I) and (*b*) (II) plotted over *d*_norm_.

**Figure 4:**
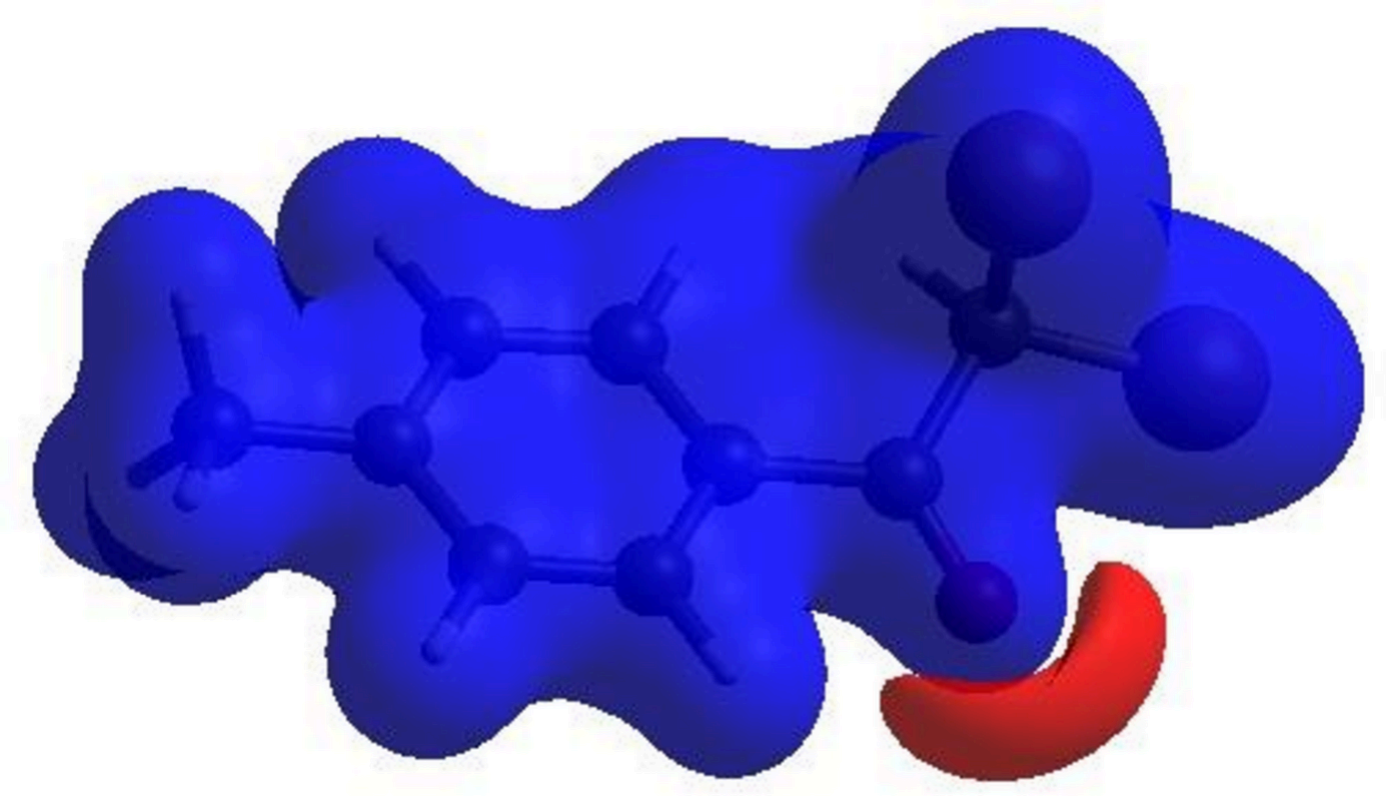
View of the three-dimensional Hirshfeld surface of compound (II) plotted over electrostatic potential energy using the STO-3 G basis set at the Hartree–Fock level of theory. Hydrogen-bond donors and acceptors are shown as the blue and red regions around the atoms corresponding to positive and negative potentials, respectively.

**Figure 5:**
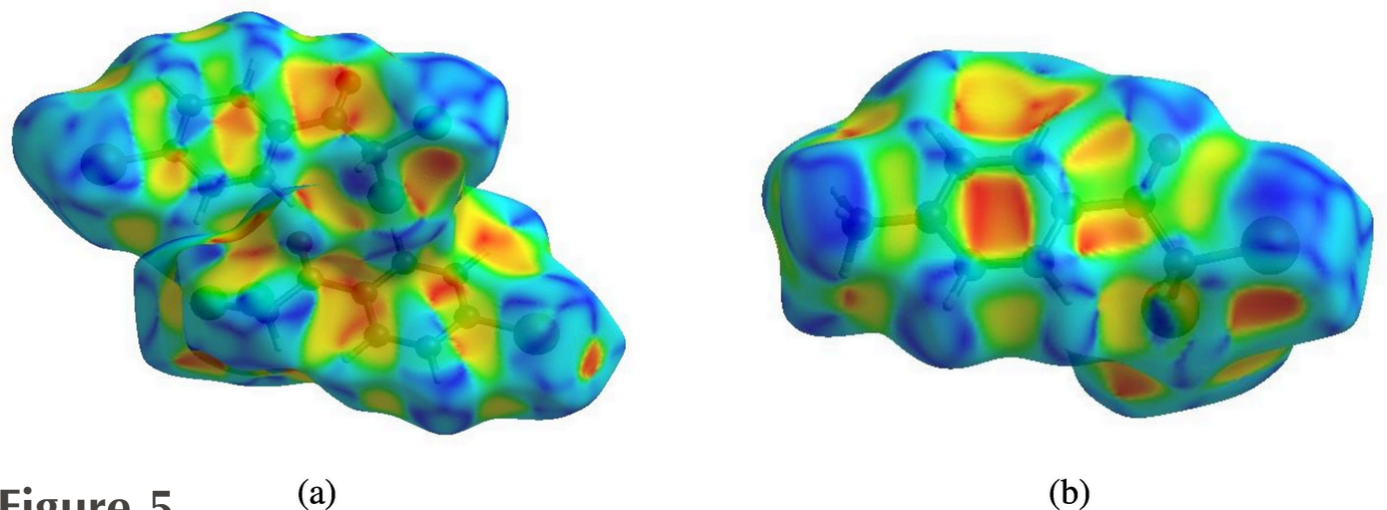
Hirshfeld surfaces of compounds (*a*) (I) and (*b*) (II) plotted over shape-index.

In order to visualize the inter­molecular inter­actions in the title compounds, Hirshfeld surface (HS) analyses (Hirshfeld, 1977 ▸; Spackman & Jayatilaka, 2009 ▸) were carried out using *Crystal Explorer 17.5* (Spackman *et al.*, 2021 ▸). In the HS plotted over *d*_norm_ (Fig. 3 ▸*a* and *b*), the white surfaces indicate contacts with distances equal to the sum of van der Waals radii, and the red and blue colours indicate distances shorter (in close contact) or longer (distinct contact) than the van der Waals radii, respectively (Venkatesan *et al.*, 2016 ▸). The bright-red spots indicate their roles as donors and/or acceptors; they also appear as blue and red regions corresponding to positive and negative potentials on the HS mapped over electrostatic potential (Spackman *et al.*, 2008 ▸; Jayatilaka *et al.*, 2005 ▸), as shown in Fig. 4 ▸ for compound (II). The π–π stacking inter­actions were further visualized by plotting the shape-index surface, which can be used to identify characteristic packing modes, in particular, planar stacking arrangements and the presence of aromatic stacking inter­actions such as C—H⋯π and π–π inter­actions. C—H⋯π inter­actions would be seen as red *p*-holes, which are related to the electron ring inter­actions between the CH groups with the centroids of the aromatic rings of neighbouring mol­ecules. Fig. 5 ▸ clearly suggests that there are no C—H⋯π inter­actions in either compound. On the other hand, the shape-index of the HS is also a tool for visualizing π–π stacking by the presence of adjacent red and blue triangles; if there are no adjacent red and/or blue triangles, then there are no π–π inter­actions. Fig. 5 ▸ clearly suggests that there are π–π inter­actions in compound (I) only.

**Figure 6:**
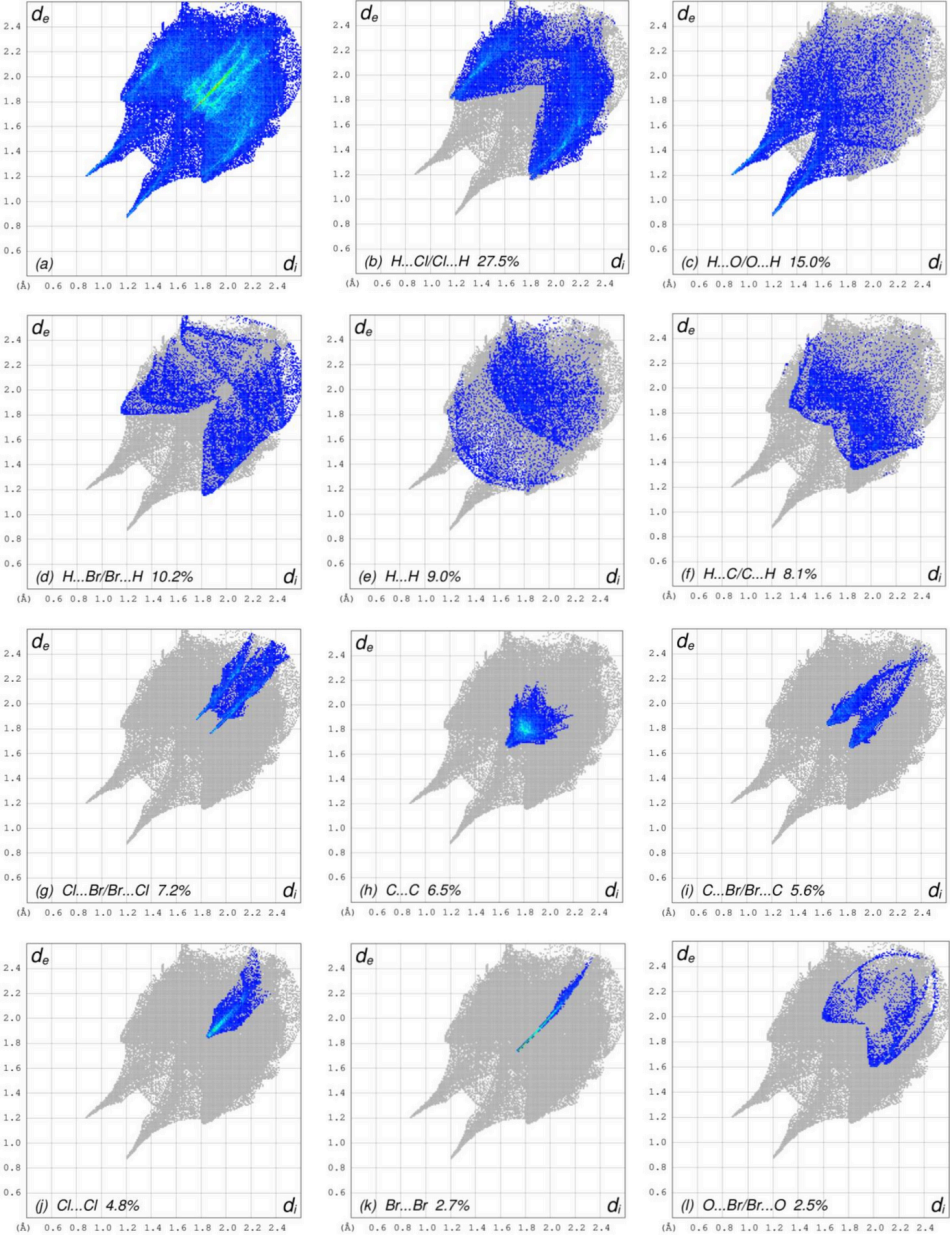
The full two-dimensional fingerprint plots for compound (I), showing (*a*) all inter­actions, and delineated into (*b*) H⋯Cl/Cl⋯H, (*c*) H⋯O/O⋯H, (*d*) H⋯Br/Br⋯H, (*e*) H⋯H, (*f*) H⋯C/C⋯H, (*g*) Cl⋯Br/Br⋯Cl, (*h*) C⋯C, (*i*) C⋯Br/Br⋯C, (*j*) Cl⋯Cl, (*k*) Br⋯Br, (*l*) O⋯Br/Br⋯O, (*m*) C⋯Cl/Cl⋯C and (*n*) O⋯Cl/Cl⋯O inter­actions. The *d*_i_ and *d_e_* values are the closest inter­nal and external distances (in Å) from given points on the Hirshfeld surface.

**Figure 7:**
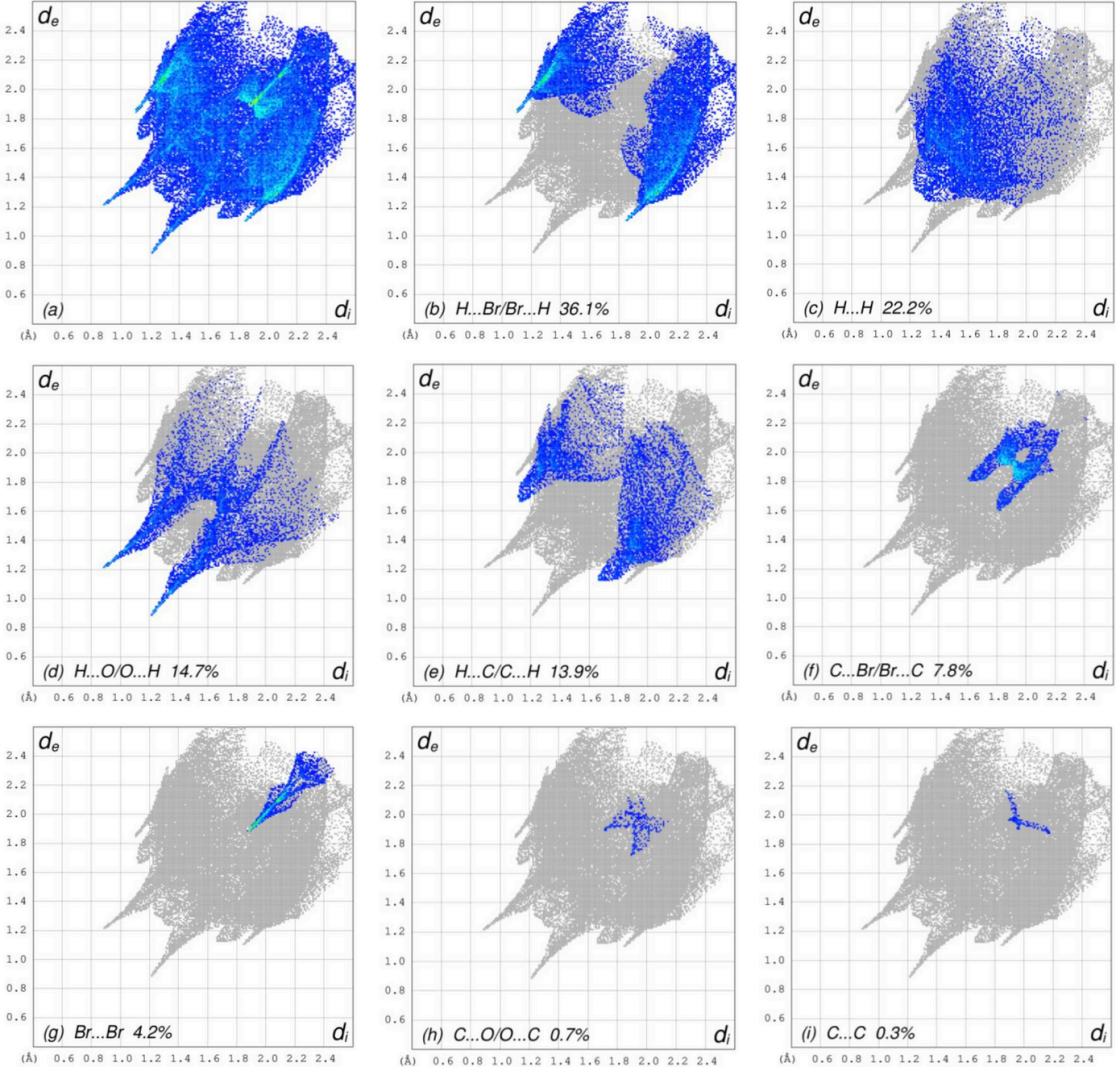
The full two-dimensional fingerprint plots for compound (II), showing (*a*) all inter­actions, and delineated into (*b*) H⋯Br/Br⋯H, (*c*) H⋯H, (*d*) H⋯O/O⋯H, (*e*) H⋯C/C⋯H, (*f*) C⋯Br/Br⋯C, (*g*) Br⋯Br, (*h*) C⋯O/O⋯C and (*i*) C⋯C inter­actions. The *d*_i_ and *d*_e_ values are the closest inter­nal and external distances (in Å) from given points on the Hirshfeld surface.

The overall two-dimensional fingerprint plots (McKinnon *et al.*, 2007 ▸) [Fig. 6 ▸*a* for (I) and Fig. 7 ▸*a* for (II)], and those delineated into H⋯Cl/Cl⋯H, H⋯O/O⋯H, H⋯Br/Br⋯H, H⋯H, H⋯C/C⋯H, Cl⋯Br/Br⋯Cl, C⋯C, C⋯Br/Br⋯C, Cl⋯Cl, Br⋯Br, O⋯Br/Br⋯O, C⋯Cl/Cl⋯C and O⋯Cl/Cl⋯O inter­actions for (I) and H⋯Br/Br⋯H, H⋯H, H⋯O/O⋯H, H⋯C/C⋯H, C⋯Br/Br⋯C, Br⋯Br, C⋯O/O⋯C and C⋯C inter­actions for (II) are illustrated in Fig. 6 ▸*b–n* and Fig. 7 ▸*b–i*, respectively, together with their relative contributions to the Hirshfeld surfaces. The most important inter­actions(Tables 3 ▸ and 4 ▸) are H⋯Cl/Cl ⋯ H for (I) and H⋯Br/Br⋯H for (II) contributing 27.5% and 36.1%, respectively, to the overall crystal packings, which are shown in Fig. 6 ▸*b* and Fig. 7 ▸*b* with the tips at *d*_e_ + *d*_i_ = 2.95 and 2.94 Å, respectively. The H⋯O/O⋯H contacts (Fig. 6 ▸*c* and Fig. 7 ▸*d*) contribute 15.0% and 14.1%, and they are viewed as the pairs of spikes with the tips at *d*_e_ + *d*_i_ = 2.08 and 2.10 Å, respectively. The H⋯Br/Br⋯H contacts in (I) (Fig. 6 ▸*d*) contribute 10.2% to the HS, and they are viewed as a pair of wings at *d*_e_ + *d*_i_ = 2.94 Å. The H⋯H contacts (Fig. 6 ▸*e* and Fig. 7 ▸*c*) have wide spreads of points, and are viewed at *d*_e_ = d_i_ = 1.40Å and 1.28 Å, respectively. In the absence of C—H⋯π inter­actions, the characteristic wings of the H⋯C/C⋯H contacts, contributing 8.1% and 13.9% to the overall crystal packings are seen in Fig. 6 ▸*f* and Fig. 7 ▸*e* with the tips at *d*_e_ + *d*_i_ = 3.24 and 2.78 Å, respectively. The tiny spikes of Cl⋯Br/Br⋯Cl for (I) (Fig. 6 ▸*g*), which contribute 7.2% to the HS are seen at *d*_e_ + *d*_i_ = 3.68 Å. The C⋯C contacts (Fig. 6 ▸*h* and Fig. 7 ▸*i*), contributing 6.5% and 0.3%, have arrow-shaped distributions of points at *d*_e_ = *d*_i_ = 1.66 Å for (I). The symmetrical pairs of C⋯Br/Br⋯C contacts (Fig. 6 ▸*i* and Fig. 7 ▸*f*) contribute 5.6% and 7.8% with the tips at *d*_e_ + *d*_i_ = 3.48 and 3.40 Å, respectively. The Cl⋯Cl contacts in (I) (Fig. 6 ▸*j*) have a bullet-shaped distribution of points with a 4.8% contribution to the HS, and the tip at *d*_e_ = *d*_i_ = 1.86 Å. The Br⋯Br contacts (Fig. 6 ▸*k* and Fig. 7 ▸*g*) contribute 2.7% and 4.2% and have a needle-shaped distributions of points, *d*_e_ = *d*_i_ = 1.74 and 1.88 Å, respectively. The O⋯Br/Br⋯O inter­actions in (I) (Fig. 6 ▸*l*) contribute 2.5% to the HS and have the tips at *d*_e_ + *d*_i_ =3.60 Å. Finally, the C⋯Cl/Cl⋯C (Fig. 6 ▸*m*), O⋯Cl/Cl⋯O (Fig. 6 ▸*n*) and C⋯O/O⋯C (Fig. 7 ▸*h*) contacts with contributions of 0.4%, 0.2% and 0.7%, respectively, have very low densities.

**Figure 8:**
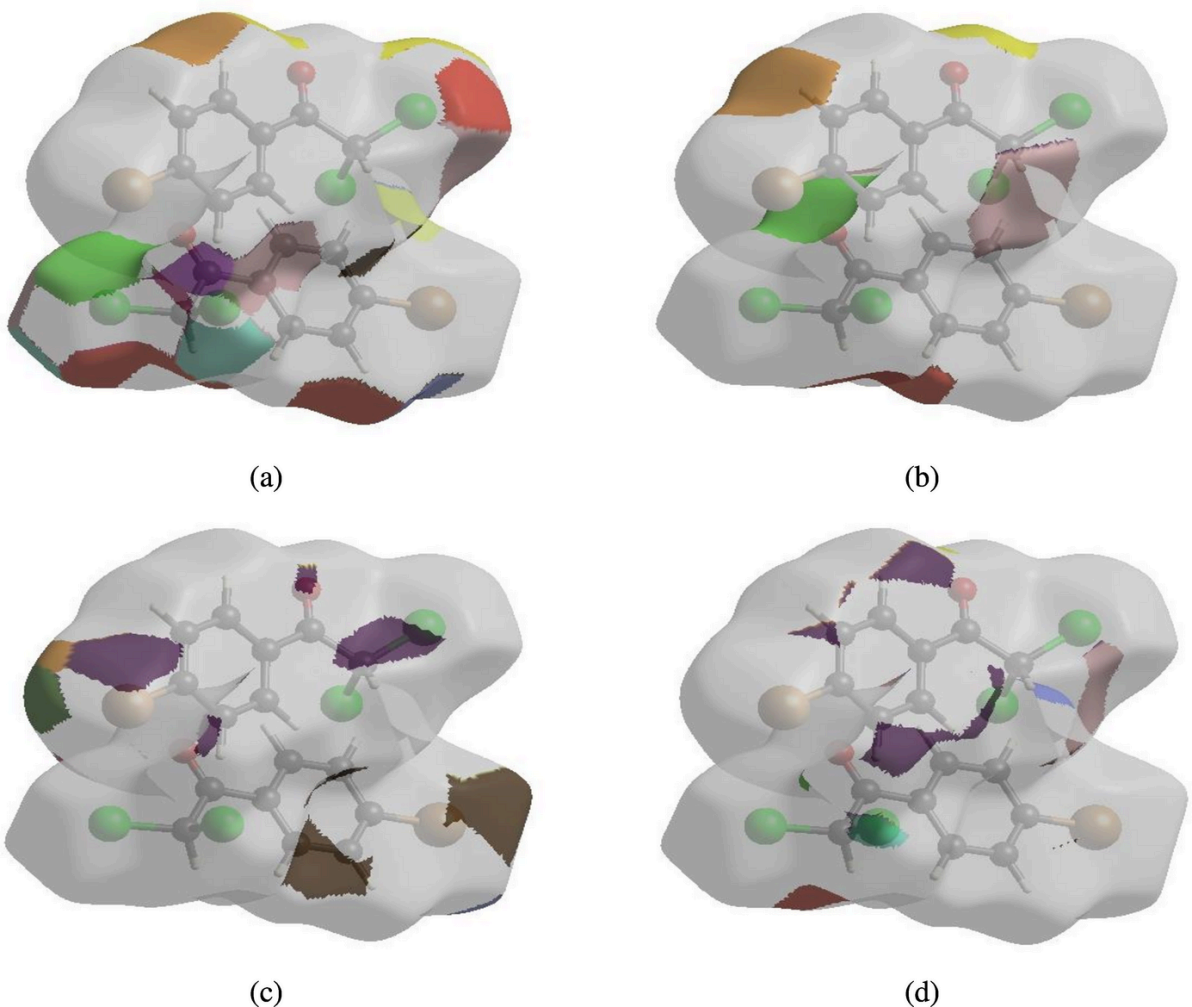
The Hirshfeld surface representations of contact patches for compound (I) plotted onto the surface for (*a*) H⋯Cl/Cl⋯H, (*b*) H⋯O/O⋯H, (*c*) H⋯Br/Br⋯H and (*d*) H⋯H inter­actions.

**Figure 9:**
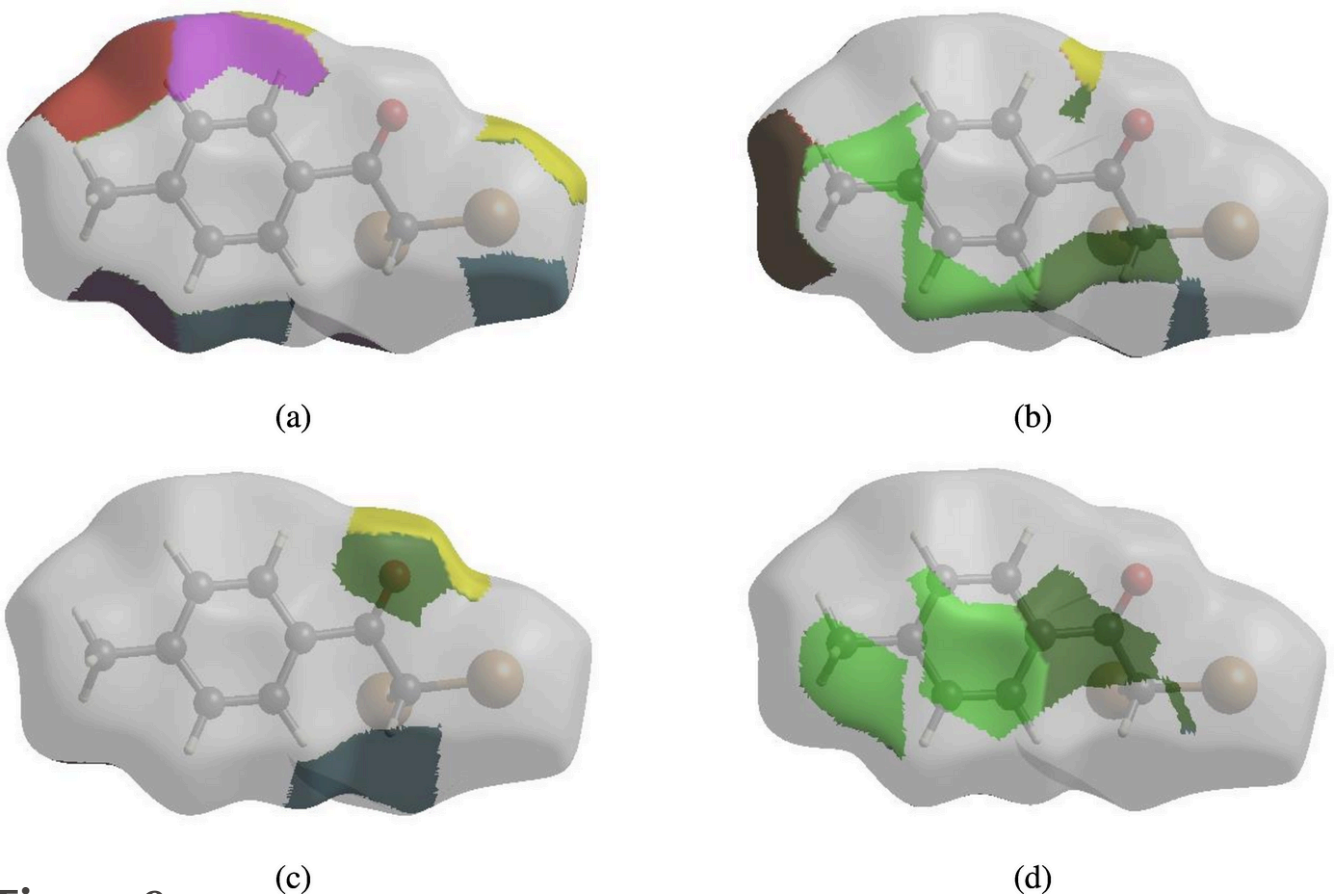
The Hirshfeld surface representations of contact patches for compound (II) plotted onto the surface for (*a*) H⋯Br/Br⋯H, (*b*) H⋯H, (*c*) H⋯O/O⋯H and (*d*) H⋯C/C⋯H inter­actions.

The nearest neighbour coordination environment of a mol­ecule can be determined from the colour patches on the HS based on how close to other mol­ecules they are. The Hirshfeld surface representations of contact patches plotted onto the surfaces are shown for the H⋯Cl/Cl⋯H, H⋯O/O⋯H, H⋯Br/Br⋯H, H⋯H and H⋯C/C⋯H inter­actions in Fig. 8 ▸*a–d* and Fig. 9 ▸*a–d* for both compounds (I) and (II), respectively.

The Hirshfeld surface analyses confirms the importance of H-atom contacts in establishing the packings. The large number of H⋯Cl/Cl⋯H, H⋯O/O⋯H, H⋯Br/Br⋯H, H⋯H and H⋯C/C⋯H inter­actions suggest that van der Waals inter­actions and hydrogen bonding play the major roles in the crystal packing (Hathwar *et al.*, 2015 ▸).

## 5. Crystal voids

**Figure 10:**
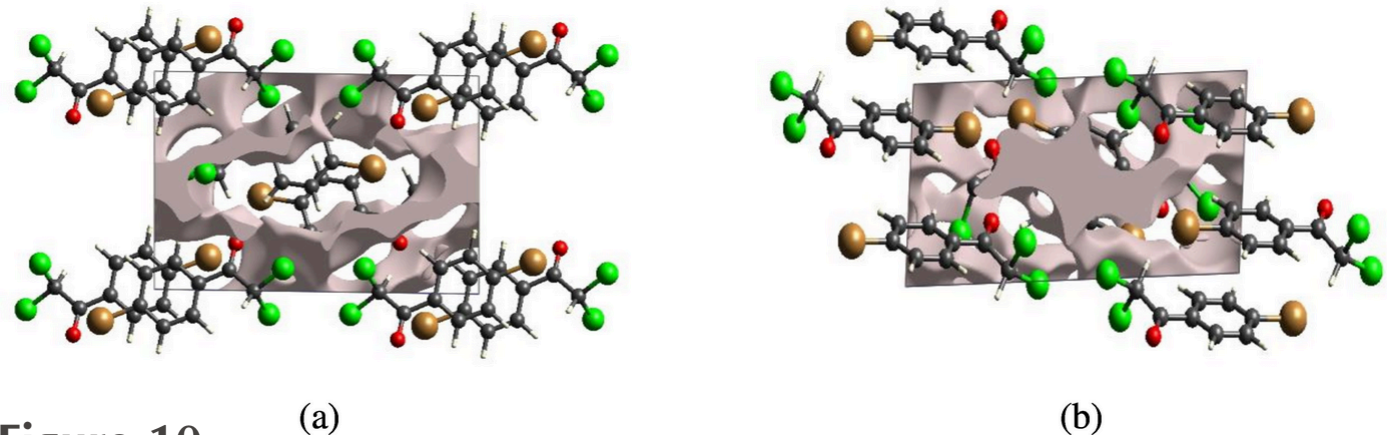
Graphical views of voids in the crystal packing of compound (I) along the (*a*) *a*-axis and (*b*) *b*-axis directions.

The strength of the crystal packing is important for determining the response to an applied mechanical force. If the crystal packing results in significant voids, the mol­ecules are not tightly packed and a small amount of applied external mechanical force may easily break the crystal. To check the mechanical stability of the crystal, a void analysis was performed by adding up the electron densities of the spherically symmetric atoms contained in the asymmetric unit (Turner *et al.*, 2011 ▸). The void surface is defined as an isosurface of the procrystal electron density and is calculated for the whole unit cell where the void surface meets the boundary of the unit cell and capping faces are generated to create an enclosed volume. The volumes of the crystal voids (Figs. 10 ▸ and 11 ▸) and the percentages of free space in the unit cells were calculated to be 111.55 Å^3^ and 12.27%, respectively, for (I) and 63.37 Å^3^ and 6.69% for (I). Thus, the crystal packings appear compact and the mechanical stability should be substantial.

## 6. Synthesis and crystallization

To a solution of 3-(4-bromo­phen­yl)-2,2-di­chloro-3-oxopropanal or 2,2-di­bromo-3-oxo-3-(*p*-tol­yl)propanal (1.00 mmol) in 20 ml of chloro­form was added di­amino­furazan (1.00 mmol) and the mixture was refluxed at 353 K for 1 h. Then, the chloro­form was evacuated under vacuum; the remaining reaction mass was added to 20 ml of diethyl ether. The precipitated *N*-(4-amino-1,2,5-oxa­diazol-3-yl)formamide (yield: 82 or 77%) was filtered off. The 1-(4-bromo­phen­yl)-2,2- di­chloro­ethan-1-one (I) or 2,2-di­bromo-1-(p-tol­yl)ethan-1-one (II) was isolated (yield: 79 or 75%) from the filtrate. (I): ^1^H NMR (300 MHz, DMSO-*d*_6_): δ = 8.20 (*d*, 2H), 7.89 (*s*, 1H), 7.44 (*d*, 2H). ^13^C NMR (151 MHz, CDCl_3_) δ = 185.60, 145.87, 129.87, 129.65, 128.75, 67.84. (II): ^1^H NMR (300 MHz, DMSO-*d*_6_): δ = 8.21 (*d*, 2H), 7.75 (*s*, 1H), 7.30 (*d*, 2H), 2.35 (*s*, 3H). ^13^C NMR (151 MHz, CDCl_3_) δ = 184.95, 141.25, 131.25, 129.49, 129.30, 51.79, 21.85. *N*-(4-Amino-1,2,5- oxa­diazol-3-yl)formamide: ^1^H NMR (300 MHz, DMSO-*d*_6_): δ = 10.40 (*bd*, 1H, NH), 8.75 (*bd*, 1H, CHO), 6.11 (*bs*, 2H, NH_2_).

## 7. Refinement

Crystal data, data collection and structure refinement details are summarized in Table 5 ▸. The C-bond hydrogen-atom positions were calculated geometrically at distances of 1.00 Å (for methine CH), 0.95 Å (for aromatic CH) and 0.98 Å (for CH_3_) and refined using a riding model by applying the constraint of *U*_iso_(H) = *k* × *U*_eq_ (C), where *k* = 1.5 for methyl H atoms and *k* = 1.2 for the other H atoms.

## Supplementary Material

Crystal structure: contains datablock(s) I, II, global. DOI: 10.1107/S205698902500012X/nx2018sup1.cif

Structure factors: contains datablock(s) I. DOI: 10.1107/S205698902500012X/nx2018Isup2.hkl

Structure factors: contains datablock(s) II. DOI: 10.1107/S205698902500012X/nx2018IIsup3.hkl

CCDC references: 2415427, 2415426

Additional supporting information:  crystallographic information; 3D view; checkCIF report

## Figures and Tables

**Figure 11:**
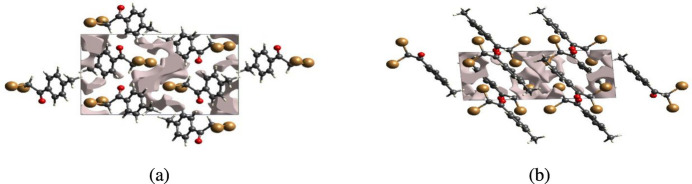
Graphical views of voids in the crystal packing of compound (II) along the (*a*) *a*-axis and (*b*) *b*-axis directions.

**Table 1:** Hydrogen-bond geometry (Å, °) for (I)

*D*—H⋯*A*	*D*—H	H⋯*A*	*D*⋯*A*	*D*—H⋯*A*
C1*A*—H1*A*⋯O2*B*^viii^	1.00	2.18	3.100 (3)	152
C1*B*—H1*B*⋯O2*A*^vi^	1.00	2.18	3.166 (3)	169
C8*A*—H8*A*⋯O2*B*^viii^	0.95	2.47	3.374 (3)	160

Symmetry codes: (vi) 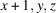; (viii) 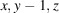.

**Table 2:** Hydrogen-bond geometry (Å, °) for (II)

*D*—H⋯*A*	*D*—H	H⋯*A*	*D*⋯*A*	*D*—H⋯*A*
C1—H1⋯O2^ii^	1.00	2.20	3.173 (4)	165
C8—H8⋯O2^ii^	0.95	2.51	3.403 (3)	157

Symmetry code: (ii) 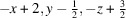.

**Table 3:** Selected interatomic distances (Å) for (I)

Br6*A*⋯Br6*B*^i^	3.4966 (4)	H5*B*⋯O2*A*^vii^	2.63
Br6*A*⋯C1*A*^ii^	3.554 (3)	O2*A*⋯H4*A*	2.50
C2*A*⋯Br6*A*^ii^	3.515 (3)	O2*B*⋯H1*A*^v^	2.18
Br6*B*⋯C2*B*^iii^	3.504 (2)	O2*B*⋯H4*B*	2.52
Br6*A*⋯H1*A*^ii^	3.03	O2*B*⋯H8*A*^v^	2.47
Cl1*A*⋯O2*A*	2.894 (2)	C6*A*⋯C8*A*^ii^	3.361 (3)
Cl1*B*⋯O2*B*	2.901 (2)	C1*A*⋯H8*A*	2.61
Cl2*B*⋯C8*B*	3.453 (3)	C1*B*⋯H8*B*	2.62
Cl2*B*⋯C4*A*	3.251 (3)	C8*A*⋯H1*A*	2.63
Cl2*B*⋯H8*B*	2.88	C8*B*⋯H1*B*	2.67
O2*A*⋯C1*B*^iv^	3.166 (3)	H1*A*⋯H8*A*	2.06
O2*B*⋯C1*A*^v^	3.100 (3)	H1*B*⋯H8*B*	2.20
H1*B*⋯O2*A*^vi^	2.18		

Symmetry codes: (i) 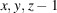; (ii) 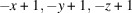; (iii) 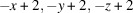; (iv) 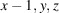; (v) 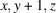; (vi) 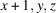; (vii) 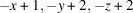.

**Table 4:** Selected interatomic distances (Å) for (II)

Br1⋯O2	3.011 (2)	H8⋯O2^ii^	2.51
C4⋯Br2^i^	3.452 (3)	C1⋯H8	2.67
C5⋯Br2^i^	3.534 (3)	C8⋯H1	2.62
C1⋯O2^ii^	3.173 (4)	H1⋯H8	2.03
O2⋯H4	2.53	H5⋯H9*C*	2.40
H1⋯O2^ii^	2.20		

Symmetry codes: (i) 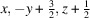; (ii) 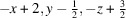.

**Table 5:** Experimental details

	(I)	(II)
Crystal data		
Chemical formula	C_8_H_5_BrCl_2_O	C_9_H_8_Br_2_O
*M*_r_	267.93	291.97
Crystal system, space group	Triclinic, *P* 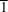	Monoclinic, *P*2_1_/*c*
Temperature (K)	100	100
*a*, *b*, *c* (Å)	7.0317 (1), 9.78938 (18), 14.4440 (3)	6.6243 (1), 9.9574 (1), 14.3804 (2)
α, β, γ (°)	87.5944 (15), 84.7254 (13), 72.0372 (14)	90, 92.520 (1), 90
*V* (Å^3^)	941.70 (3)	947.63 (2)
*Z*	4	4
Radiation type	Cu *K*α	Cu *K*α
μ (mm^−1^)	10.75	10.43
Crystal size (mm)	0.35 × 0.21 × 0.16	0.22 × 0.16 × 0.12

Data collection		
Diffractometer	XtaLAB Synergy, Dualflex, HyPix	XtaLAB Synergy, Dualflex, HyPix
Absorption correction	Gaussian (*CrysAlis PRO*; Rigaku OD, 2024 ▸)	Gaussian (CrysAlisPr; Rigaku OD, 2024 ▸)
*T*_min_, *T*_max_	0.169, 1.000	0.198, 0.680
No. of measured, independent and observed [*I* > 2σ(*I*)] reflections	24768, 4027, 3992	12954, 2075, 2046
*R*_int_	0.041	0.031
(sin θ/λ)_max_ (Å^−1^)	0.638	0.640

Refinement		
*R*[*F*^2^ > 2σ(*F*^2^)], *wR*(*F*^2^), *S*	0.031, 0.085, 1.10	0.027, 0.071, 1.08
No. of reflections	4027	2075
No. of parameters	218	111
H-atom treatment	H-atom parameters constrained	H-atom parameters constrained
Δρ_max_, Δρ_min_ (e Å^−3^)	0.63, −0.71	0.61, −0.51

Computer programs: *CrysAlis PRO* (Rigaku OD, 2024 ▸), *SHELXT2018/2* (Sheldrick, 2015*a* ▸), *SHELXL2018/3* (Sheldrick, 2015*b* ▸) and *OLEX2* (Dolomanov *et al.*, 2009 ▸).

## supplementary crystallographic information

### 1-(4-Bromophenyl)-2,2-dichloroethan-1-one (I) Crystal data

**Table:**

C_8_H_5_BrCl_2_O	*Z* = 4
*M**_r_* = 267.93	*F*(000) = 520
Triclinic, *P*1	*D*_x_ = 1.890 Mg m^−^^3^
*a* = 7.0317 (1) Å	Cu *K*α radiation, λ = 1.54184 Å
*b* = 9.78938 (18) Å	Cell parameters from 19038 reflections
*c* = 14.4440 (3) Å	θ = 4.7–79.0°
α = 87.5944 (15)°	µ = 10.75 mm^−^^1^
β = 84.7254 (13)°	*T* = 100 K
γ = 72.0372 (14)°	Prism, colorless
*V* = 941.70 (3) Å^3^	0.35 × 0.21 × 0.16 mm

### 1-(4-Bromophenyl)-2,2-dichloroethan-1-one (I) Data collection

**Table:**

XtaLAB Synergy, Dualflex, HyPix diffractometer	4027 independent reflections
Radiation source: micro-focus sealed X-ray tube, PhotonJet (Cu) X-ray Source	3992 reflections with *I* > 2σ(*I*)
Mirror monochromator	*R*_int_ = 0.041
Detector resolution: 10.0000 pixels mm^-1^	θ_max_ = 79.7°, θ_min_ = 3.1°
ω scans	*h* = −8→8
Absorption correction: gaussian (CrysAlisPro; Rigaku OD, 2024)	*k* = −12→12
*T*_min_ = 0.169, *T*_max_ = 1.000	*l* = −18→18
24768 measured reflections	

### 1-(4-Bromophenyl)-2,2-dichloroethan-1-one (I) Refinement

**Table:**

Refinement on *F*^2^	Secondary atom site location: difference Fourier map
Least-squares matrix: full	Hydrogen site location: inferred from neighbouring sites
*R*[*F*^2^ > 2σ(*F*^2^)] = 0.031	H-atom parameters constrained
*wR*(*F*^2^) = 0.085	*w* = 1/[σ^2^(*F*_o_^2^) + (0.048*P*)^2^ + 1.1375*P*] where *P* = (*F*_o_^2^ + 2*F*_c_^2^)/3
*S* = 1.10	(Δ/σ)_max_ = 0.003
4027 reflections	Δρ_max_ = 0.63 e Å^−^^3^
218 parameters	Δρ_min_ = −0.70 e Å^−^^3^
0 restraints	Extinction correction: *SHELXL2018/3* (Sheldrick, 2015b), Fc^*^=kFc[1+0.001xFc^2^λ^3^/sin(2θ)]^-1/4^
Primary atom site location: dual	Extinction coefficient: 0.0022 (2)

### 1-(4-Bromophenyl)-2,2-dichloroethan-1-one (I) Special details

**Table:**

Geometry. All esds (except the esd in the dihedral angle between two l.s. planes) are estimated using the full covariance matrix. The cell esds are taken into account individually in the estimation of esds in distances, angles and torsion angles; correlations between esds in cell parameters are only used when they are defined by crystal symmetry. An approximate (isotropic) treatment of cell esds is used for estimating esds involving l.s. planes.

### 1-(4-Bromophenyl)-2,2-dichloroethan-1-one (I) Fractional atomic coordinates and isotropic or equivalent isotropic displacement parameters (Å^2^)

**Table:**

	*x*	*y*	*z*	*U*_iso_*/*U*_eq_	
Br6A	0.81615 (4)	0.56770 (3)	0.32820 (2)	0.02069 (10)	
Cl1A	0.33348 (10)	0.55329 (7)	0.89875 (4)	0.02549 (15)	
Cl2A	0.74935 (10)	0.53706 (8)	0.84399 (4)	0.02822 (15)	
O2A	0.3354 (3)	0.7709 (2)	0.75703 (14)	0.0284 (4)	
C1A	0.5180 (4)	0.5343 (3)	0.80452 (16)	0.0175 (4)	
H1A	0.538721	0.440813	0.773449	0.021*	
C2A	0.4551 (4)	0.6576 (3)	0.73301 (17)	0.0175 (4)	
C3A	0.5482 (3)	0.6324 (2)	0.63618 (16)	0.0149 (4)	
C4A	0.4938 (4)	0.7454 (3)	0.57111 (18)	0.0191 (5)	
H4A	0.402128	0.835143	0.590281	0.023*	
C5A	0.5718 (4)	0.7276 (3)	0.47965 (18)	0.0194 (5)	
H5A	0.533876	0.803928	0.435599	0.023*	
C6A	0.7077 (3)	0.5952 (3)	0.45312 (16)	0.0161 (4)	
C7A	0.7639 (3)	0.4816 (2)	0.51561 (16)	0.0156 (4)	
H7A	0.856155	0.392344	0.495928	0.019*	
C8A	0.6835 (3)	0.4999 (2)	0.60758 (16)	0.0141 (4)	
H8A	0.720183	0.422604	0.651044	0.017*	
Br6B	0.76334 (4)	0.85069 (3)	1.16685 (2)	0.02310 (10)	
Cl1B	1.01787 (9)	1.10238 (6)	0.60398 (4)	0.02204 (14)	
Cl2B	0.80524 (11)	0.89180 (7)	0.64724 (5)	0.03046 (16)	
O2B	0.7017 (3)	1.21469 (19)	0.74989 (12)	0.0218 (4)	
C1B	0.9394 (4)	0.9975 (2)	0.69302 (16)	0.0170 (4)	
H1B	1.059740	0.932687	0.721540	0.020*	
C2B	0.7983 (3)	1.0930 (2)	0.76903 (16)	0.0152 (4)	
C3B	0.7895 (3)	1.0296 (2)	0.86393 (16)	0.0147 (4)	
C4B	0.7014 (4)	1.1233 (3)	0.93713 (17)	0.0180 (5)	
H4B	0.646312	1.223144	0.924605	0.022*	
C5B	0.6938 (4)	1.0718 (3)	1.02798 (17)	0.0204 (5)	
H5B	0.636309	1.135395	1.078017	0.024*	
C6B	0.7723 (4)	0.9248 (3)	1.04401 (16)	0.0177 (4)	
C7B	0.8588 (4)	0.8292 (3)	0.97239 (18)	0.0207 (5)	
H7B	0.910242	0.729156	0.985011	0.025*	
C8B	0.8688 (4)	0.8826 (3)	0.88192 (17)	0.0192 (5)	
H8B	0.929655	0.818923	0.832274	0.023*	

### 1-(4-Bromophenyl)-2,2-dichloroethan-1-one (I) Atomic displacement parameters (Å^2^)

**Table:**

	*U*^11^	*U*^22^	*U*^33^	*U*^12^	*U*^13^	*U*^23^
Br6A	0.02097 (15)	0.03124 (16)	0.01216 (14)	−0.01176 (11)	−0.00107 (9)	0.00224 (10)
Cl1A	0.0275 (3)	0.0262 (3)	0.0176 (3)	−0.0025 (2)	0.0063 (2)	−0.0035 (2)
Cl2A	0.0248 (3)	0.0411 (4)	0.0190 (3)	−0.0092 (3)	−0.0068 (2)	0.0002 (2)
O2A	0.0356 (10)	0.0181 (9)	0.0214 (9)	0.0064 (8)	0.0004 (8)	−0.0053 (7)
C1A	0.0193 (11)	0.0176 (11)	0.0118 (10)	−0.0005 (8)	0.0017 (8)	−0.0028 (8)
C2A	0.0202 (11)	0.0148 (10)	0.0164 (11)	−0.0026 (8)	−0.0035 (9)	−0.0028 (8)
C3A	0.0160 (10)	0.0132 (10)	0.0159 (10)	−0.0046 (8)	−0.0028 (8)	−0.0025 (8)
C4A	0.0211 (11)	0.0152 (10)	0.0208 (12)	−0.0046 (9)	−0.0036 (9)	−0.0002 (9)
C5A	0.0214 (11)	0.0163 (11)	0.0207 (12)	−0.0053 (9)	−0.0054 (9)	0.0035 (9)
C6A	0.0163 (10)	0.0218 (11)	0.0127 (10)	−0.0098 (9)	−0.0008 (8)	−0.0007 (9)
C7A	0.0150 (10)	0.0170 (10)	0.0148 (11)	−0.0044 (8)	−0.0019 (8)	−0.0010 (8)
C8A	0.0138 (10)	0.0144 (10)	0.0141 (10)	−0.0039 (8)	−0.0027 (8)	0.0000 (8)
Br6B	0.02356 (16)	0.03377 (17)	0.01465 (15)	−0.01308 (11)	−0.00342 (10)	0.00666 (10)
Cl1B	0.0306 (3)	0.0205 (3)	0.0144 (3)	−0.0082 (2)	0.0030 (2)	0.0001 (2)
Cl2B	0.0472 (4)	0.0293 (3)	0.0233 (3)	−0.0233 (3)	−0.0023 (3)	−0.0082 (2)
O2B	0.0258 (9)	0.0166 (8)	0.0184 (8)	−0.0011 (7)	0.0011 (7)	0.0020 (7)
C1B	0.0239 (11)	0.0152 (10)	0.0119 (10)	−0.0061 (9)	−0.0008 (9)	−0.0012 (8)
C2B	0.0165 (10)	0.0146 (10)	0.0147 (10)	−0.0046 (8)	−0.0020 (8)	−0.0020 (8)
C3B	0.0140 (10)	0.0165 (10)	0.0143 (10)	−0.0053 (8)	−0.0024 (8)	0.0001 (8)
C4B	0.0219 (11)	0.0152 (10)	0.0181 (11)	−0.0071 (9)	−0.0018 (9)	−0.0015 (9)
C5B	0.0240 (12)	0.0225 (12)	0.0162 (11)	−0.0095 (9)	0.0004 (9)	−0.0042 (9)
C6B	0.0187 (11)	0.0250 (12)	0.0119 (10)	−0.0101 (9)	−0.0038 (8)	0.0034 (9)
C7B	0.0226 (12)	0.0182 (11)	0.0190 (12)	−0.0041 (9)	0.0000 (9)	0.0044 (9)
C8B	0.0199 (11)	0.0169 (11)	0.0172 (11)	−0.0011 (9)	0.0016 (9)	−0.0020 (9)

### 1-(4-Bromophenyl)-2,2-dichloroethan-1-one (I) Geometric parameters (Å, º)

**Table:**

Br6A—C6A	1.890 (2)	Br6B—C6B	1.892 (2)
Cl1A—C1A	1.766 (2)	Cl1B—C1B	1.766 (2)
Cl2A—C1A	1.781 (3)	Cl2B—C1B	1.781 (2)
O2A—C2A	1.209 (3)	O2B—C2B	1.210 (3)
C1A—H1A	1.0000	C1B—H1B	1.0000
C1A—C2A	1.540 (3)	C1B—C2B	1.542 (3)
C2A—C3A	1.486 (3)	C2B—C3B	1.485 (3)
C3A—C4A	1.404 (3)	C3B—C4B	1.398 (3)
C3A—C8A	1.403 (3)	C3B—C8B	1.396 (3)
C4A—H4A	0.9500	C4B—H4B	0.9500
C4A—C5A	1.380 (4)	C4B—C5B	1.388 (4)
C5A—H5A	0.9500	C5B—H5B	0.9500
C5A—C6A	1.397 (3)	C5B—C6B	1.390 (4)
C6A—C7A	1.385 (3)	C6B—C7B	1.389 (3)
C7A—H7A	0.9500	C7B—H7B	0.9500
C7A—C8A	1.391 (3)	C7B—C8B	1.391 (3)
C8A—H8A	0.9500	C8B—H8B	0.9500
			
Br6A···Br6B^i^	3.4966 (4)	H5B···O2A^vii^	2.63
Br6A···C1A^ii^	3.554 (3)	O2A···H4A	2.50
C2A···Br6A^ii^	3.515 (3)	O2B···H1A^v^	2.18
Br6B···C2B^iii^	3.504 (2)	O2B···H4B	2.52
Br6A···H1A^ii^	3.03	O2B···H8A^v^	2.47
Cl1A···O2A	2.894 (2)	C6A···C8A^ii^	3.361 (3)
Cl1B···O2B	2.901 (2)	C1A···H8A	2.61
Cl2B···C8B	3.453 (3)	C1B···H8B	2.62
Cl2B···C4A	3.251 (3)	C8A···H1A	2.63
Cl2B···H8B	2.88	C8B···H1B	2.67
O2A···C1B^iv^	3.166 (3)	H1A···H8A	2.06
O2B···C1A^v^	3.100 (3)	H1B···H8B	2.20
H1B···O2A^vi^	2.18		
			
Cl1A—C1A—Cl2A	110.59 (13)	Cl1B—C1B—Cl2B	110.50 (13)
Cl1A—C1A—H1A	109.1	Cl1B—C1B—H1B	109.2
Cl2A—C1A—H1A	109.1	Cl2B—C1B—H1B	109.2
C2A—C1A—Cl1A	111.28 (16)	C2B—C1B—Cl1B	111.22 (16)
C2A—C1A—Cl2A	107.49 (17)	C2B—C1B—Cl2B	107.41 (16)
C2A—C1A—H1A	109.1	C2B—C1B—H1B	109.2
O2A—C2A—C1A	119.8 (2)	O2B—C2B—C1B	119.7 (2)
O2A—C2A—C3A	122.4 (2)	O2B—C2B—C3B	122.9 (2)
C3A—C2A—C1A	117.9 (2)	C3B—C2B—C1B	117.4 (2)
C4A—C3A—C2A	118.0 (2)	C4B—C3B—C2B	117.6 (2)
C8A—C3A—C2A	122.5 (2)	C8B—C3B—C2B	122.5 (2)
C8A—C3A—C4A	119.5 (2)	C8B—C3B—C4B	119.9 (2)
C3A—C4A—H4A	119.6	C3B—C4B—H4B	119.7
C5A—C4A—C3A	120.8 (2)	C5B—C4B—C3B	120.6 (2)
C5A—C4A—H4A	119.6	C5B—C4B—H4B	119.7
C4A—C5A—H5A	120.7	C4B—C5B—H5B	120.8
C4A—C5A—C6A	118.7 (2)	C4B—C5B—C6B	118.4 (2)
C6A—C5A—H5A	120.7	C6B—C5B—H5B	120.8
C5A—C6A—Br6A	119.57 (18)	C5B—C6B—Br6B	119.59 (18)
C7A—C6A—Br6A	118.54 (18)	C7B—C6B—Br6B	118.29 (18)
C7A—C6A—C5A	121.9 (2)	C7B—C6B—C5B	122.1 (2)
C6A—C7A—H7A	120.4	C6B—C7B—H7B	120.6
C6A—C7A—C8A	119.1 (2)	C6B—C7B—C8B	118.9 (2)
C8A—C7A—H7A	120.4	C8B—C7B—H7B	120.6
C3A—C8A—H8A	120.0	C3B—C8B—H8B	119.9
C7A—C8A—C3A	120.0 (2)	C7B—C8B—C3B	120.1 (2)
C7A—C8A—H8A	120.0	C7B—C8B—H8B	119.9
			
Br6A—C6A—C7A—C8A	−179.28 (17)	Br6B—C6B—C7B—C8B	−179.14 (19)
Cl1A—C1A—C2A—O2A	23.6 (3)	Cl1B—C1B—C2B—O2B	25.4 (3)
Cl1A—C1A—C2A—C3A	−157.33 (17)	Cl1B—C1B—C2B—C3B	−154.12 (17)
Cl2A—C1A—C2A—O2A	−97.6 (2)	Cl2B—C1B—C2B—O2B	−95.6 (2)
Cl2A—C1A—C2A—C3A	81.4 (2)	Cl2B—C1B—C2B—C3B	84.8 (2)
O2A—C2A—C3A—C4A	0.1 (4)	O2B—C2B—C3B—C4B	−14.4 (3)
O2A—C2A—C3A—C8A	−178.4 (2)	O2B—C2B—C3B—C8B	167.0 (2)
C1A—C2A—C3A—C4A	−178.9 (2)	C1B—C2B—C3B—C4B	165.1 (2)
C1A—C2A—C3A—C8A	2.6 (3)	C1B—C2B—C3B—C8B	−13.5 (3)
C2A—C3A—C4A—C5A	−178.6 (2)	C2B—C3B—C4B—C5B	−178.0 (2)
C2A—C3A—C8A—C7A	179.0 (2)	C2B—C3B—C8B—C7B	179.1 (2)
C3A—C4A—C5A—C6A	−0.6 (4)	C3B—C4B—C5B—C6B	−1.2 (4)
C4A—C3A—C8A—C7A	0.6 (3)	C4B—C3B—C8B—C7B	0.5 (4)
C4A—C5A—C6A—Br6A	179.75 (18)	C4B—C5B—C6B—Br6B	−179.69 (18)
C4A—C5A—C6A—C7A	0.8 (4)	C4B—C5B—C6B—C7B	0.5 (4)
C5A—C6A—C7A—C8A	−0.3 (3)	C5B—C6B—C7B—C8B	0.6 (4)
C6A—C7A—C8A—C3A	−0.4 (3)	C6B—C7B—C8B—C3B	−1.2 (4)
C8A—C3A—C4A—C5A	−0.1 (4)	C8B—C3B—C4B—C5B	0.7 (4)

Symmetry codes: (i) *x*, *y*, *z*−1; (ii) −*x*+1, −*y*+1, −*z*+1; (iii) −*x*+2, −*y*+2, −*z*+2; (iv) *x*−1, *y*, *z*; (v) *x*, *y*+1, *z*; (vi) *x*+1, *y*, *z*; (vii) −*x*+1, −*y*+2, −*z*+2.

### 1-(4-Bromophenyl)-2,2-dichloroethan-1-one (I) Hydrogen-bond geometry (Å, º)

**Table:**

*D*—H···*A*	*D*—H	H···*A*	*D*···*A*	*D*—H···*A*
C1*A*—H1*A*···O2*B*^viii^	1.00	2.18	3.100 (3)	152
C1*B*—H1*B*···O2*A*^vi^	1.00	2.18	3.166 (3)	169
C8*A*—H8*A*···O2*B*^viii^	0.95	2.47	3.374 (3)	160

Symmetry codes: (vi) *x*+1, *y*, *z*; (viii) *x*, *y*−1, *z*.

### 2,2-Dibromo-1-(4-methylphenyl)ethan-1-one (II) Crystal data

**Table:**

C_9_H_8_Br_2_O	*F*(000) = 560
*M**_r_* = 291.97	*D*_x_ = 2.047 Mg m^−^^3^
Monoclinic, *P*2_1_/*c*	Cu *K*α radiation, λ = 1.54184 Å
*a* = 6.6243 (1) Å	Cell parameters from 8712 reflections
*b* = 9.9574 (1) Å	θ = 4.4–80.2°
*c* = 14.3804 (2) Å	µ = 10.43 mm^−^^1^
β = 92.520 (1)°	*T* = 100 K
*V* = 947.63 (2) Å^3^	Prism, colorless
*Z* = 4	0.22 × 0.16 × 0.12 mm

### 2,2-Dibromo-1-(4-methylphenyl)ethan-1-one (II) Data collection

**Table:**

XtaLAB Synergy, Dualflex, HyPix diffractometer	2075 independent reflections
Radiation source: micro-focus sealed X-ray tube, PhotonJet (Cu) X-ray Source	2046 reflections with *I* > 2σ(*I*)
Mirror monochromator	*R*_int_ = 0.031
Detector resolution: 10.0000 pixels mm^-1^	θ_max_ = 80.6°, θ_min_ = 5.4°
ω scans	*h* = −8→6
Absorption correction: gaussian (CrysAlisPr; Rigaku OD, 2024)	*k* = −12→12
*T*_min_ = 0.198, *T*_max_ = 0.680	*l* = −18→18
12954 measured reflections	

### 2,2-Dibromo-1-(4-methylphenyl)ethan-1-one (II) Refinement

**Table:**

Refinement on *F*^2^	Secondary atom site location: difference Fourier map
Least-squares matrix: full	Hydrogen site location: inferred from neighbouring sites
*R*[*F*^2^ > 2σ(*F*^2^)] = 0.027	H-atom parameters constrained
*wR*(*F*^2^) = 0.071	*w* = 1/[σ^2^(*F*_o_^2^) + (0.0353*P*)^2^ + 1.8407*P*] where *P* = (*F*_o_^2^ + 2*F*_c_^2^)/3
*S* = 1.08	(Δ/σ)_max_ = 0.001
2075 reflections	Δρ_max_ = 0.61 e Å^−^^3^
111 parameters	Δρ_min_ = −0.51 e Å^−^^3^
0 restraints	Extinction correction: *SHELXL2018/3* (Sheldrick, 2015b), Fc^*^=kFc[1+0.001xFc^2^λ^3^/sin(2θ)]^-1/4^
Primary atom site location: dual	Extinction coefficient: 0.00143 (13)

### 2,2-Dibromo-1-(4-methylphenyl)ethan-1-one (II) Special details

**Table:**

Geometry. All esds (except the esd in the dihedral angle between two l.s. planes) are estimated using the full covariance matrix. The cell esds are taken into account individually in the estimation of esds in distances, angles and torsion angles; correlations between esds in cell parameters are only used when they are defined by crystal symmetry. An approximate (isotropic) treatment of cell esds is used for estimating esds involving l.s. planes.

### 2,2-Dibromo-1-(4-methylphenyl)ethan-1-one (II) Fractional atomic coordinates and isotropic or equivalent isotropic displacement parameters (Å^2^)

**Table:**

	*x*	*y*	*z*	*U*_iso_*/*U*_eq_	
Br1	1.23695 (4)	0.66709 (3)	0.63489 (2)	0.01960 (11)	
Br2	0.77764 (4)	0.64644 (3)	0.55958 (2)	0.02528 (12)	
O2	0.9676 (3)	0.8317 (2)	0.75385 (16)	0.0251 (4)	
C1	0.9651 (4)	0.6269 (3)	0.66762 (18)	0.0169 (5)	
H1	0.959154	0.532581	0.691175	0.020*	
C2	0.8893 (4)	0.7229 (3)	0.74202 (18)	0.0163 (5)	
C3	0.7113 (4)	0.6791 (3)	0.79372 (19)	0.0181 (5)	
C4	0.5987 (4)	0.7784 (3)	0.83659 (18)	0.0186 (5)	
H4	0.639502	0.869684	0.833713	0.022*	
C5	0.4273 (4)	0.7438 (3)	0.88335 (18)	0.0195 (5)	
H5	0.350118	0.812055	0.911211	0.023*	
C6	0.3669 (4)	0.6097 (3)	0.88997 (18)	0.0185 (5)	
C7	0.4849 (4)	0.5111 (3)	0.84988 (19)	0.0195 (5)	
H7	0.449098	0.419262	0.856196	0.023*	
C8	0.6538 (4)	0.5447 (3)	0.80095 (18)	0.0186 (5)	
H8	0.730070	0.476472	0.772465	0.022*	
C9	0.1787 (4)	0.5707 (3)	0.9386 (2)	0.0236 (6)	
H9A	0.216416	0.531296	0.999369	0.035*	
H9B	0.102049	0.504843	0.900725	0.035*	
H9C	0.095251	0.650586	0.947391	0.035*	

### 2,2-Dibromo-1-(4-methylphenyl)ethan-1-one (II) Atomic displacement parameters (Å^2^)

**Table:**

	*U*^11^	*U*^22^	*U*^33^	*U*^12^	*U*^13^	*U*^23^
Br1	0.01651 (16)	0.02134 (17)	0.02127 (17)	−0.00045 (9)	0.00428 (10)	0.00094 (10)
Br2	0.02045 (17)	0.03458 (19)	0.02068 (17)	−0.00176 (11)	−0.00073 (11)	−0.00414 (11)
O2	0.0244 (10)	0.0201 (10)	0.0314 (11)	−0.0065 (8)	0.0095 (8)	−0.0060 (8)
C1	0.0136 (11)	0.0193 (12)	0.0179 (12)	−0.0003 (9)	0.0025 (9)	0.0003 (10)
C2	0.0161 (11)	0.0152 (12)	0.0176 (11)	−0.0002 (9)	0.0015 (9)	0.0014 (9)
C3	0.0164 (12)	0.0177 (12)	0.0203 (12)	−0.0002 (10)	0.0010 (10)	0.0007 (10)
C4	0.0190 (12)	0.0169 (12)	0.0198 (12)	−0.0005 (9)	−0.0001 (9)	0.0001 (10)
C5	0.0201 (12)	0.0218 (13)	0.0167 (11)	0.0045 (10)	0.0022 (9)	−0.0031 (10)
C6	0.0176 (12)	0.0248 (13)	0.0132 (11)	−0.0005 (10)	0.0013 (9)	−0.0001 (10)
C7	0.0182 (12)	0.0193 (12)	0.0210 (12)	−0.0011 (10)	0.0007 (10)	0.0008 (10)
C8	0.0187 (12)	0.0177 (13)	0.0196 (12)	0.0005 (9)	0.0030 (9)	0.0008 (10)
C9	0.0199 (12)	0.0287 (15)	0.0228 (13)	−0.0025 (11)	0.0053 (10)	−0.0001 (11)

### 2,2-Dibromo-1-(4-methylphenyl)ethan-1-one (II) Geometric parameters (Å, º)

**Table:**

Br1—C1	1.923 (3)	C5—H5	0.9500
Br2—C1	1.955 (3)	C5—C6	1.398 (4)
O2—C2	1.210 (3)	C6—C7	1.396 (4)
C1—H1	1.0000	C6—C9	1.507 (4)
C1—C2	1.535 (4)	C7—H7	0.9500
C2—C3	1.487 (4)	C7—C8	1.389 (4)
C3—C4	1.398 (4)	C8—H8	0.9500
C3—C8	1.396 (4)	C9—H9A	0.9800
C4—H4	0.9500	C9—H9B	0.9800
C4—C5	1.388 (4)	C9—H9C	0.9800
			
Br1···O2	3.011 (2)	H8···O2^ii^	2.51
C4···Br2^i^	3.452 (3)	C1···H8	2.67
C5···Br2^i^	3.534 (3)	C8···H1	2.62
C1···O2^ii^	3.173 (4)	H1···H8	2.03
O2···H4	2.53	H5···H9C	2.40
H1···O2^ii^	2.20		
			
Br1—C1—Br2	110.71 (13)	C6—C5—H5	119.6
Br1—C1—H1	109.2	C5—C6—C9	121.5 (2)
Br2—C1—H1	109.2	C7—C6—C5	118.4 (2)
C2—C1—Br1	112.31 (18)	C7—C6—C9	120.0 (3)
C2—C1—Br2	106.03 (17)	C6—C7—H7	119.4
C2—C1—H1	109.2	C8—C7—C6	121.2 (3)
O2—C2—C1	120.3 (2)	C8—C7—H7	119.4
O2—C2—C3	122.5 (2)	C3—C8—H8	120.1
C3—C2—C1	117.2 (2)	C7—C8—C3	119.8 (3)
C4—C3—C2	117.6 (2)	C7—C8—H8	120.1
C8—C3—C2	122.9 (2)	C6—C9—H9A	109.5
C8—C3—C4	119.5 (2)	C6—C9—H9B	109.5
C3—C4—H4	119.9	C6—C9—H9C	109.5
C5—C4—C3	120.1 (3)	H9A—C9—H9B	109.5
C5—C4—H4	119.9	H9A—C9—H9C	109.5
C4—C5—H5	119.6	H9B—C9—H9C	109.5
C4—C5—C6	120.9 (2)		
			
Br1—C1—C2—O2	21.6 (3)	C2—C3—C8—C7	−179.6 (2)
Br1—C1—C2—C3	−161.77 (18)	C3—C4—C5—C6	1.3 (4)
Br2—C1—C2—O2	−99.5 (3)	C4—C3—C8—C7	0.5 (4)
Br2—C1—C2—C3	77.2 (2)	C4—C5—C6—C7	1.1 (4)
O2—C2—C3—C4	18.2 (4)	C4—C5—C6—C9	−178.6 (2)
O2—C2—C3—C8	−161.7 (3)	C5—C6—C7—C8	−2.8 (4)
C1—C2—C3—C4	−158.4 (2)	C6—C7—C8—C3	2.0 (4)
C1—C2—C3—C8	21.7 (4)	C8—C3—C4—C5	−2.1 (4)
C2—C3—C4—C5	178.0 (2)	C9—C6—C7—C8	177.0 (2)

Symmetry codes: (i) *x*, −*y*+3/2, *z*+1/2; (ii) −*x*+2, *y*−1/2, −*z*+3/2.

### 2,2-Dibromo-1-(4-methylphenyl)ethan-1-one (II) Hydrogen-bond geometry (Å, º)

**Table:**

*D*—H···*A*	*D*—H	H···*A*	*D*···*A*	*D*—H···*A*
C1—H1···O2^ii^	1.00	2.20	3.173 (4)	165
C8—H8···O2^ii^	0.95	2.51	3.403 (3)	157

Symmetry code: (ii) −*x*+2, *y*−1/2, −*z*+3/2.

## References

[bb1] Dolomanov, O. V., Bourhis, L. J., Gildea, R. J., Howard, J. A. K. & Puschmann, H. (2009). *J. Appl. Cryst.* **42**, 339–341.

[bb4] Erian, A. W., Sherif, S. M. & Gaber, H. M. (2003). *Molecules*, **8**, 793–865.

[bb5] Gurbanov, A. V., Kuznetsov, M. L., Karmakar, A., Aliyeva, V. A., Mahmudov, K. T. & Pombeiro, A. J. L. (2022). *Dalton Trans.* **51**, 1019–1031. 10.1039/d1dt03755b 34935834

[bb6] Guseinov, F. I., Pistsov, M. F., Malinnikov, V. M., Lavrova, O. M., Movsumzade, E. M. & Kustov, L. M. (2020). *Mendeleev Commun.* **30**, 674–675.

[bb7] Guseinov, F. I., Pistsov, M. F., Movsumzade, E. M., Kustov, L. M., Tafeenko, V. A., Chernyshev, V. V., Gurbanov, A. V., Mahmudov, K. T. & Pombeiro, A. (2017). *Crystals*, **7**, 327.

[bb8] Guseinov, F. N., Burangulova, R. N., Mukhamedzyanova, E. F., Strunin, B. P., Sinyashin, O. G., Litvinov, I. A. & Gubaidullin, A. T. (2006). *Chem. Heterocycl. Compd.* **42**, 943–947.

[bb9] Guseinov, F. N., Ovsyannikov, V. O., Shuvalova, E. V., Kustov, L. M., Kobrakov, K. I., Samigullina, A. I. & Mahmudov, K. T. (2024). *New J. Chem.* **48**, 12869–12872.

[bb10] Hathwar, V. R., Sist, M., Jørgensen, M. R. V., Mamakhel, A. H., Wang, X., Hoffmann, C. M., Sugimoto, K., Overgaard, J. & Iversen, B. B. (2015). *IUCrJ*, **2**, 563–574. 10.1107/S2052252515012130 PMC4547824 26306198

[bb11] Hirshfeld, H. L. (1977). *Theor. Chim. Acta*, **44**, 129–138.

[bb12] Jayatilaka, D., Grimwood, D. J., Lee, A., Lemay, A., Russel, A. J., Taylor, C., Wolff, S. K., Cassam-Chenai, P. & Whitton, A. (2005). *TONTO - A System for Computational Chemistry.* Available at: *http://hirshfeldsurface.net/*.

[bb13] Khalilov, A. N., Cisterna, J., Cárdenas, A., Tuzun, B., Erkan, S., Gurbanov, A. V. & Brito, I. (2024). *J. Mol. Struct.* **1313**, 138652.

[bb14] Ma, Z., Mahmudov, K. T., Aliyeva, V. A., Gurbanov, A. V., Guedes da Silva, M. F. C. & Pombeiro, A. J. L. (2021). *Coord. Chem. Rev.* **437**, 213859.

[bb15] Mahmoudi, G., Zaręba, J. K., Gurbanov, A. V., Bauzá, A., Zubkov, F. I., Kubicki, M., Stilinović, V., Kinzhybalo, V. & Frontera, A. (2017). *Eur. J. Inorg. Chem.* pp. 4763–4772.

[bb16] McKinnon, J. J., Jayatilaka, D. & Spackman, M. A. (2007). *Chem. Commun.* pp. 3814–3816. 10.1039/b704980c 18217656

[bb17] Mizar, A., Guedes da Silva, M. F. C., Kopylovich, M. N., Mukherjee, S., Mahmudov, K. T. & Pombeiro, A. J. L. (2012). *Eur. J. Inorg. Chem.* **2012**, 2305–2313.

[bb18] Rigaku OD (2024). *CrysAlis PRO*. Rigaku Oxford Diffraction, Yarnton, England.

[bb19] Sheldrick, G. M. (2015*a*). *Acta Cryst.* A**71**, 3–8.

[bb20] Sheldrick, G. M. (2015*b*). *Acta Cryst.* C**71**, 3–8.

[bb24] Spackman, M. A. & Jayatilaka, D. (2009). *CrystEngComm*, **11**, 19–32.

[bb25] Spackman, M. A., McKinnon, J. J. & Jayatilaca, D. (2008). *CrystEngComm*, **10**, 377–388.

[bb26] Spackman, P. R., Turner, M. J., McKinnon, J. J., Wolff, S. K., Grimwood, D. J., Jayatilaka, D. & Spackman, M. A. (2021). *J. Appl. Cryst.* **54**, 1006–1011. 10.1107/S1600576721002910 PMC8202033 34188619

[bb27] Turner, M. J., McKinnon, J. J., Jayatilaka, D. & Spackman, M. A. (2011). *CrystEngComm*, **13**, 1804–1813.

[bb28] Venkatesan, P., Thamotharan, S., Ilangovan, A., Liang, H. & Sundius, T. (2016). *Spectrochim. Acta A Mol. Biomol. Spectrosc.* **153**, 625–636. 10.1016/j.saa.2015.09.002 26452098

